# SARS-CoV-2 intra-host evolution during acute infection in COVID-19 patients

**DOI:** 10.3389/fmicb.2026.1794039

**Published:** 2026-04-28

**Authors:** Carla della Ventura, Annalisa Bergna, Cosmin Lucian Ciubotariu, Mirko Liturri, Claudia Conflitti, Mario Corbellino, Spinello Antinori, Agostino Riva, Gianguglielmo Zehender, Alessia Lai

**Affiliations:** 1 Department of Biomedical and Clinical Sciences, University of Milan, Milan, Italy; 2 III Division of Infectious Diseases, ASST Fatebenefratelli Sacco, Luigi Sacco Hospital, Milan, Italy; 3 National PhD Programme in One Health Approaches to Infectious Diseases and Life Science Research, Department of Public Health, Experimental and Forensic Medicine, University of Pavia, Pavia, Italy

**Keywords:** intra-host evolution, mutational pattern, number of mutations, SARS-CoV-2, viral variants

## Abstract

**Introduction:**

The intra-host evolution represents the mechanism causing the continued emergence of new, highly divergent SARS-CoV-2 variants. The aim of this work was to investigate the intra-host evolution to ass the conditions associated with the acquisition of new viral mutations.

**Methods:**

Whole genome sequences of 58 cases of COVID-19 with 2 or more consecutive positive swabs were analyzed. Variant calling and minority mutations analysis were performed using Nextclade and Stanford Coronavirus Antiviral & Resistance Database.

**Results:**

Fifty-seven subjects were hospitalized for a median of 23 days, and 12 died (20.7%) after a median of 25 days. 78.9% (45/58) were vaccinated and 22.4% (13/58) received more than one treatment for SARS-CoV-2 infection. None reported previous SARS-CoV-2 infection. Negativization period showed longer intervals in BQ.1 infections (38.5 days) and shorter in BA.1 infections (16.5 days). Comparing the first positive swab to the second swab (T1 and T2), 36.7 and 38.8% decreased/increased number of mutations while 24.5% maintained the same number. Subjects with constant numbers of mutations maintained the same pattern while 57% of the acquisitions and losses were confirmed compared to the previous timepoint. Comparing the second to the third swab (T2 and T3), 66.7 and 33.3% of strains increased or maintained the same number of mutations, respectively. 75% of subjects with constant number of mutations maintained the same pattern. 63% of the acquisitions were confirmed. In T1 vs. T2 the acquisitions were prevalent in the S gene and the losses in ORF1a, in T2 vs. T3 the opposite was observed. Subjects with cardiovascular disease showed a significantly higher acquisition of mutations over time.

**Discussion:**

Our data suggested a mechanism with different steps of fitness selection of SARS-CoV-2 mutations and highlighted the presence of highly divergent intra-patient variants within 3 weeks of infection, regardless of treatment, confirming data from other studies showing that new viral variants can also emerge during acute infections.

## Graphical abstract

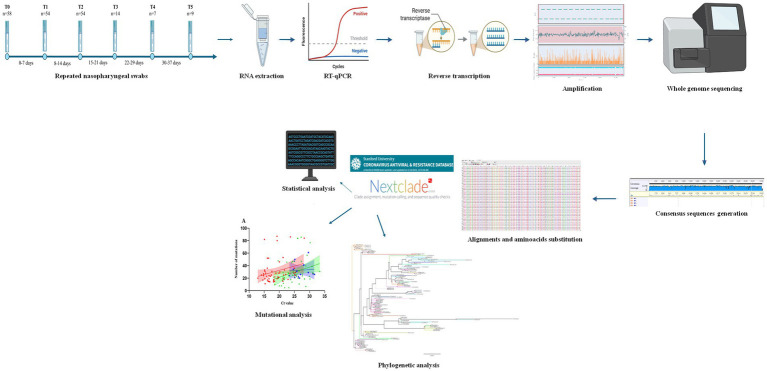

## 1 Introduction

The intra-host evolution is a dynamic process depending on several factors, including the host characteristics, the immune response and the characteristics of the virus itself. Several studies demonstrated that in patients with prolonged COVID-19 infection and/or a compromised immune system, the virus continues to evolve accumulating mutations. This increase in genetic diversity can lead to the emergence of new viral variants (Raglow et al., 2024; Avanzato et al., 2020).

The rate of evolution of SARS-CoV-2 is considered moderate, estimated around 1.19–1.31 × 10^–3^ substitutions per site per year (s/s/y) (Li et al., 2020), tending to increase mainly due to the low fidelity and the fast elongation activity of viral RNA-dependent RNA polymerase (RdRp) (Sun et al., 2020). RNA polymerase also exhibits low nucleotide insertion fidelity, resulting in a higher probability of errors inclusion during viral genome replication (V'Kovski et al., 2021; Denison et al., 2011). In response to the low replication fidelity, coronaviruses present an exonuclease, nsp14, that works to proofread and exclude mismatched nucleotides during the replication process (Jones et al., 2022). The remaining errors generate different types of mutations (point mutations, insertions or deletions) that have been associated with better adaptation of the virus to the host environment. Some of these mutations, however, may affect the virus’ ability to bind cell receptors Angiotensin-Converting Enzyme 2 (ACE2), alter transmissibility or immune escape (Harvey et al., 2021; Papanikolaou et al., 2022). Furthermore, mutations in the RdRp gene can affect the efficiency of replication/transcription machinery, leading to a more error-prone mutant, increasing viral genetic diversity and enabling the virus to adapt to various selective pressures. Since lower fidelity is often linked to faster replication, these mutations may also result in higher viral titers within host cells (Eskier et al., 2020).

The mutations acquired during the infection give rise to a diverse viral population, known as a ‘quasi-species’, in which each virion exhibits slight genetic variation, yet all variants originate from a common ancestral sequence. This mechanism, extensively studied in HIV and other RNA viruses, leads to genetic variability within the viral population, playing a role in maintaining viral diversity within host (Dudouet et al., 2022; Smet et al., 2025). The diversity within the viral population is an adaptive mechanism of the virus that enables it to adapt more rapidly to the pressure of immunological or pharmacological conditions, favoring a longer and/or more severe infection (Sun et al., 2020; Carabelli et al., 2023). The selection of a viral population is driven by the host immune response according to a process of natural selection. The presence of neutralizing antibodies and T-cells, that recognize the virus by binding to specific antigens, can select viral variants able to escape this immune surveillance (Zhang, 2024; Garcia-Beltran et al., 2021). The innate immune response, mediated by interferons, cytokines and innate cells can induce error mechanisms during viral replication and thereby contribute to the emergence of viral variants that are more resistant to the immune response (Thorne et al., 2022). Most of these mutations occur in the Spike protein, particularly in the RBD (Receptor Binding Domain) portion, the ACE2 receptor binding site involved in virus entry into host cells. This protein is the main target for vaccines and most neutralizing antibodies (Papanikolaou et al., 2022).

Less frequently, mutations have also been observed in the N (Nucleocapsid) protein and open reading frame (ORF) 3a genes, which give the virus the ability to evade or regulate the host immune response (Issa et al., 2020; Johnson et al., 2022).

The emergence of new viral variants is therefore due to a pattern conjunction of variability at the population and the intra-host levels, which are products of selective, stochastic and spatiotemporal processes. Moreover, the high levels of transmission in the community allows the virus to replicate rapidly and extensively, leading to the creation of great genetic diversity within the viral population. This process can also promote the emergence of more resistant or infectious variants. The emergence of highly divergent lineages such as omicron has shown that SARS-CoV-2 can rapidly evolve to evade vaccine-induced immunity, indicating the need for their frequent updating (Braun et al., 2021; Minkoff and tenOever, 2023). Recent study highlighted an increasing in genomic mutation rate and a shift from purifying to neutral selection after the introduction of vaccination and in more recent periods when most of the population presented SARS-CoV-2 immunity and Omicron lineages prevailed (Gupta et al., 2024). Different works speculated that the origin of diverse lineages is the result of intra-host evolution due to the suboptimal immune responses followed by onward transmission, particularly in immunocompromised individuals (Gonzalez-Reiche et al., 2023; Quaranta et al., 2022).

Following the hypothesis suggested by Ghafari et al. (2024) and Chaguza et al. (2023), according to which a group of people or a single patient with long-term infection could potentially be a larger source of divergent variants than expected, we studied the entire viral genome of isolates obtained from 58 nasopharyngeal swabs, sampled at different times during the course of the disease in order to assess the dynamics of the SARS-CoV-2 intra-host genetic variability and mutations.

## 2 Materials and methods

### 2.1 Samples collection

**Figure 1:**
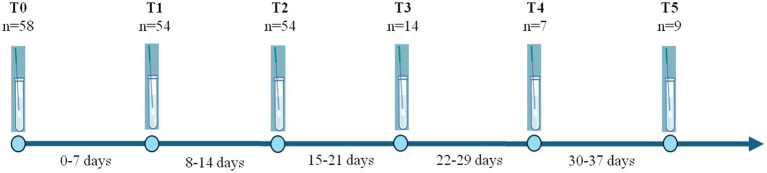
Schematic representation of the samples collected at the different time points analyzed in the study.

Study was conducted at Luigi Sacco Hospital in Milan in the period 2021–2023. Cases of COVID-19 with 2 or more longitudinally positive SARS-CoV-2 swabs, collected at intervals of at least 7 days from the first positive swab (T0), were analysed, named consecutively T1, T2, T3, T4 and T5, when available (Figure 1). Main results of the present work focused on T1, T2, and T3 due to the large number of samples (T1 *vs.* T2 = 51; T2 *vs.* T3 = 12; T3 *vs.* T4 = 3; T4 *vs.* T5 = 7).

All participants gave informed consent for the study participation. Clinical information and demographic data for each patient were collected by hospital staff and physicians. All data used in this study were previously anonymized as required by the Italian Data Protection Code (Legislative Decree 196/2003) and the general authorizations issued by the Data Protection Authority. The study was conducted in compliance with Good Clinical Practice and the Declaration of Helsinki and approved by the Sacco Hospital ethical committee (protocol n. 47866, 9 September 2020).

### 2.2 Whole genome amplification

Viral RNA was extracted from 200 μL of nasopharyngeal swabs with a QIAamp Viral RNA kit (Qiagen, Venlo, Netherlands), eluted in 50 μL of RNAse free water and used as the template for RT-PCR to ascertain the cycle threshold (Ct). The qPCR was performed using Luna^®^ Universal One-Step RT-qPCR (New England BioLabs, Ipswich, MA) and SARS-CoV-2 (2019-nCoV) CDC qPCR Probe Assay (Integrated DNA Technologies, Coralville, IA) kits. Only samples with a Ct value ≤33 were included. SARS-CoV-2 amplicons were obtained using primers pools developed by ARTIC Network (Artic V5.3.2 NCOV-2019 Panel, IDT, Coralville, IA^1^), purified with AMPure XP beads (Beckman Coulter, Indianapolis, IN) and checked for the correct size using TapeStation 200 (Agilent Technologies, Santa Clara, CA). Amplicon libraries were prepared using Illumina DNA Prep and IDT ILMN DNA/RNA Index kit (Illumina, SanDiego, CA). Library concentration was determined with the Invitrogen Quant-iT Picogreen dsDNA assay (Fisher Thermo Scientific, Waltham, MA). Resulting libraries were normalized and pooled for subsequent sequencing on Illumina MiSeq platform using a 2 × 200 cycle paired-end sequencing protocol. The instrument generally maintains a low error rate ranging from 0.1–1 × 10^−2^ per base sequenced. Reads were mapped and aligned to the SARS-CoV-2 reference genome available on the Global Initiative on Sharing Avian Influenza Data (GISAID) website^2^ (accession ID: EPI_ISL_406800). Consensus sequences were obtained using Geneious Prime software (v.2025.1.1; Biomatters, Auckland, New Zealand). SARS-CoV-2 sequences were classified using the Pangolin COVID-19 Lineage Assigner tool v.4.3.1^3^ and Nextclade v.3.15.3^4^ (Tarkowski et al., 2021). No resequencing was necessary. Complete genomes of T1 have been submitted to GISAID.

### 2.3 Variant calling and minority mutations analysis

Single-nucleotide variants (SNVs) were detected using Nextclade v.3.15.3 that identify nucleotide and amino acid substitutions relative to the reference genome Wuhan-Hu-1 (NC_045512.2).

The software outputs detailed per-sequence reports including substitutions, deletions, insertions, and frameshifts across annotated genomic regions. These data were exported and used for downstream comparative analyses of mutation patterns (Aksamentov et al., 2021). A custom bioinformatic pipeline that categorizes detected mutations by viral genes was also used. The mutation rate has been calculated considering SNVs refers to the length of the reference genome sequence (29,903 nucleotides). Detailed mutational profiling was focused on the time points with the largest number of available samples (T1–T3) as previously described. Data from T4 and T5 were not included in the mutational analysis.

To better characterize the within-host viral diversity, consensus sequences were exported from Geneious Prime software by applying variant frequency thresholds, from 25 to 95%, enabling detection of minority variants, not retained in standard (majority-rule) consensus sequence. All the variants present in each sample at a frequency ≥5%, with a variant position coverage depth >400x were considered.

The identification of mutations in specific viral proteins within each gene, as well as their potential functional impact related to antiviral resistance or immune escape, was performed using the Stanford Coronavirus Antiviral & Resistance Database.^5^

### 2.4 Genetic distances

To assess the degree of divergence among sequences within the dataset, genetic distances were calculated using MEGA12.^6^ The analysis was performed using both the number-of-differences and p-distance methods, with statistical support provided by 100 replicates using the bootstrap algorithm.

Distances were computed for within-patient comparisons across different follow-up timepoints, and between samples with different SARS-CoV-2 variants.

In addition, the ratio of synonymous to non-synonymous mutations was calculated to evaluate the selective pressure on the viral genome. If dN/dS ratio exceeds unity (dN/dS > 1), the mutations are said to be occurring under positive selection which promotes the accumulation of beneficial mutations, whereas if dN/dS ratio is below unity (dN/dS < 1), the mutations are said to be occurring under negative selection promoting mutations that are favoring selective removal of deleterious alleles.

### 2.5 Phylogenetic analysis

Multiple sequence alignment was made using Nextclade and the alignment was manually edited by the BioEdit v. 7.2.6.1 program. Phylogenetic trees were obtained by Maximum Likelihood approach using IQ-TREE v2.2.1^7^ with 1,000 bootstrap replicates and visualized using FigTree (v1.4.4). TN + F + R2 (Tamura–Nei with empirical base frequencies and FreeRate heterogeneity, 2 categories) model was selected by ModelFinder for phylogenetic analysis.

### 2.6 Statistical analysis

No statistical method was used to predetermine samples size; however, statistic refers to comparisons with largest availability of samples. Baseline characteristics were expressed as absolute and percentage frequencies for categorical variables, and median with interquartile range [IQR] for continuous variables.

Differences among mutations were assessed by using the ANOVA test, or its non-parametric version (Wilcoxon Mann Whitney and Kruskal Wallis tests), for continuous variables. For categorical variables, the χ^2^ test or the Fisher’s exact test, as appropriate, were used. For all hypotheses tested, significance was reached for *p-values* < 0.05.

All the analyses have been conducted with R software, v4.3.3.

Data visualization and statistical analysis were performed using GraphPad Prism 9. Spearman’s correlation analysis was conducted to evaluate the relationship between Ct value (Baden et al., 2020) and number of mutations. The correlation was evaluated separately for both total mutations and specific mutations within the Spike protein for each group (T1, T2, and T3). The shaded areas represent the 95% confidence intervals of the regression lines.

## 3 Results

### 3.1 Patient characteristics

Fifty-eight patients were enrolled in the study; the median age was 83 years (IQR: 73–88), and the majority of subjects were males (55.2%, *n* = 32). All but one of the subjects were hospitalized for a median of 23 days (IQR: 16–32), and 12 died (20.7%) after a median of 25 days (IQR: 19–34) of hospitalization. None reported previous SARS-CoV-2 infection, 78.9% (*n* = 45) had been vaccinated and about half had received 3 doses (46.7%, *n* = 21).

Seven subjects (12.3%) had no comorbidities and therefore received no treatment, while 22.4% (*n* = 13) received more than one treatment for SARS-CoV-2 infection. Sixty percent (23/38) of the subjects with monotherapy had received Remdesivir and 46% (6/13) of those with polytherapy had received Remdesivir + Nirmatrelvir/Ritonavir. The large majority of subjects (*n* = 32, 65.3%) presented at least two comorbidities (Table 1). Four subjects presented coinfections, 2 HIV and 2 HBV (Supplementary Table 1).

**Table 1:** Characteristics of studied population.

Characteristics	
Study population	58
Sex n(%)	
M	32 (55.2)
F	26 (44.8)
Median age [IQR]	83 [73–88]
Hospitalized *n* (%)	57 (98.3)
Non hospitalized *n* (%)	1 (1.7)
Death *n* (%)	12 (20.7)
Median days of hospitalization [IQR]	23 [16–32]
Median days between hospitalization and death [IQR]	25 [19–34]
Median days of negativization [IQR]	18 [15–23]
Median Ct value [IQR]	18.3 [16–21.2]
Vaccinated *n* (%)	45 (78.9)
2 doses *n* (%)	9 (20)
3 doses *n* (%)	21 (46.7)
4 doses *n* (%)	15 (33.3)
Median of doses [IQR]	3.0 [3.0, 4.0]
Unvaccinated *n* (%)	12 (21.1)
Previous documented infection	0
Treatment	
Monotherapy n (%)	38 (65.5)
Polytherapy n (%)	13 (22.4)
No	7 (12.1)
Comorbidity	
No *n* (%)	7 (12.3)
Yes *n* (%)	50 (87.7)
At least 1 *n* (%)	49 (98)
2 *n* (%)	32 (65.3)
3 *n* (%)	12 (24.5)
More than 3 *n* (%)	1 (2)
No of comorbidity (median [IQR])	3.0 [2.0, 4.0]
Missing	2
High comorbidity (>3)	25 (44.6)
Missing	2
Immunosuppression	27 (47.4)
Missing	1
Variant	
Delta *n* (%)	4 (6.9)
BA.1 *n* (%)	6 (10.3)
BA.2 *n* (%)	14 (24.1)
BA.4 *n* (%)	2 (3.4)
BA.5 *n* (%)	8 (13.8)
BQ *n* (%)	5 (8.6)
XBB *n* (%)	18 (31)
XCH.1 *n* (%)	1 (1.7)

In accordance with the study period, several viral variants were detected. The majority of subject were XBB recombinants (31%, *n* = 18) and BA.2 lineage (24.1%, *n* = 14) (Table 1).

Median days of negativization was 18 days (IQR: 15–23) (Table 1). Stratifying according to viral variant, negativization time showed significant differences, with longer intervals in BQ (38.5 days, IQR:26–51) and shorter in BA.1 infections (16.5 days, IQR:15–18) (*p = 0.04*) (Supplementary Figure 1). Median Ct values was significantly (*p < 0.00001*) different among T1-T5 groups (T1 = 18.8, IQR:16.1–21.2; T2 = 24.9, IQR:21.8–27.6; T3 = 27.1, IQR:25.1–29.2, T4 = 24, IQR:22.7–27, T5 = 26.9 IQR: 24.5–27).

Breakthrough infection was observed after a median of 261 days (IQR: 158–419) from the last administration of the SARS-CoV-2 vaccine with no differences among viral variants.

Half of subjects who died carried XBB sub lineage (*n* = 6), while 3 were BA.5, 2 BA.2 and one BA.4. The main cause of death (*n* = 10) was SARS-CoV-2 pneumonia frequently associated with bacterial or fungal superinfection (*n* = 7).

No differences were observed in time to clear-up the infection period when considering different therapies or monotherapy compared to combination therapy for SARS-CoV-2 infection or no treatment. Moreover, no significant differences were present in vaccinated *vs.* non vaccinated subjects also considering number of received doses.

### 3.2 Genetic distances

Only sequences with a high coverage value (mean 2556.17; min 0 – max 14413.6) were used to analyze all the reads and to find minority mutations. The mean Q30 quality score was 93.4 (min 90.5-max 95) and the coverage of 98–100% of the genome was obtained. Genetic distance analysis was conducted across all time points (T1 to T5). The overall mean number of differences was 91.8 (standard error, S.E. 3.66) with a *p-distance* of 0.003 (S.E. 0.0). Within each point, the group with the highest average number of differences was T5 (106.3, S.E. 7.12) with a *p-distance* of 0.004 (S.E. 0.0).

The comparison among time points confirmed that T5 showed the highest number of nucleotide differences when compared with all other time points T5 *vs.* T1: 110.4 (S.E. 4.56) differences, *p-distance* 0.00394 (S.E. 0.0); T5 *vs.* T2: 103.4 (S.E. 4.48) differences*, p-distance* 0.00357 (S.E. 0.0); T5 *vs.* T3: 102 (S.E. 4.46) differences, *p-distance* 0.00351 (S.E. 0.0); T5 *vs.* T4: 83.5 (S.E. 4.28) differences, *p-distance* 0.00288 (S.E. 0.0).

Genetic distance analysis at time points T1 and T2 showed an overall mean genetic distance across all sequences of 92.5 number of differences (S.E. 4.039), with a corresponding *p-distance* of 0.003 (S.E. 0.0). Considering viral variants, the highest average genetic distance was observed in BA.1 variant at both time points. Specifically, BA.1 showed a within-group average number of differences of 159.7 (*p-distance* = 0.008) at T1 and 71.5 (*p-distance* = 0.003) at T2.

When comparing the same variant in the two time points, the same trend was observed. Variant BA.1 showed a higher number of differences, with an average of 113.44 (S.E. 5.25) and a corresponding *p-distance* of 0.00511 (S.E. 0.0).

The ratio of nonsynonymous (dN) to synonymous (dS) mutations was calculated using the same groupings applied in the genetic distance analysis. Within each sampling time point, the within-group average dN/dS ratios did not exceed 1, indicating a purifying selective pressure. Between groups, a dN/dS ratio > 1 was observed only comparing T4 and T5 (0.00258/0.00233 = 1.1), indicating a diversifying.

In the comparison between time points T1 and T2 stratified by viral variants, none of the within-group average dN/dS ratios exceeded 1. Comparing the same viral variant across different time points, a dN/dS ratio >1 was observed only between T1 and T2 for the BQ variant.

### 3.3 Mutations and variability analysis

A total of 8,084 single-nucleotide variants (SNV) were observed in all times analyzed, with different numbers of substitutions detected at each time point, 3,923, 3,258, 903, 692 and 937 in T1, T2, T3, T4 and T5, respectively.

Proportion of SNV significantly decreased in studied time point from 13.1 to 3% (*p < 0.00001*) according to the reduction of aminoacidic mutations (from 16.6 to 4.7%, *p < 0.00001*; mean 30; min 5 max 87; mean 30, min 10 max 86; mean 33, min 20 max 62; mean 23, min 8 max 32; mean 27, min 10 max 38 in T1, T2, T3, T4 and T5 respectively). The mutations have not resulted in changes in lineage classification.

Globally, the frequency of transitions was higher than that of transversions (C > T = 536 *vs.* T > A = 215).

Nine hundred and six amino acid mutations were observed in all time points, mainly nonsynonymous (96.6%).

**Figure 2:**
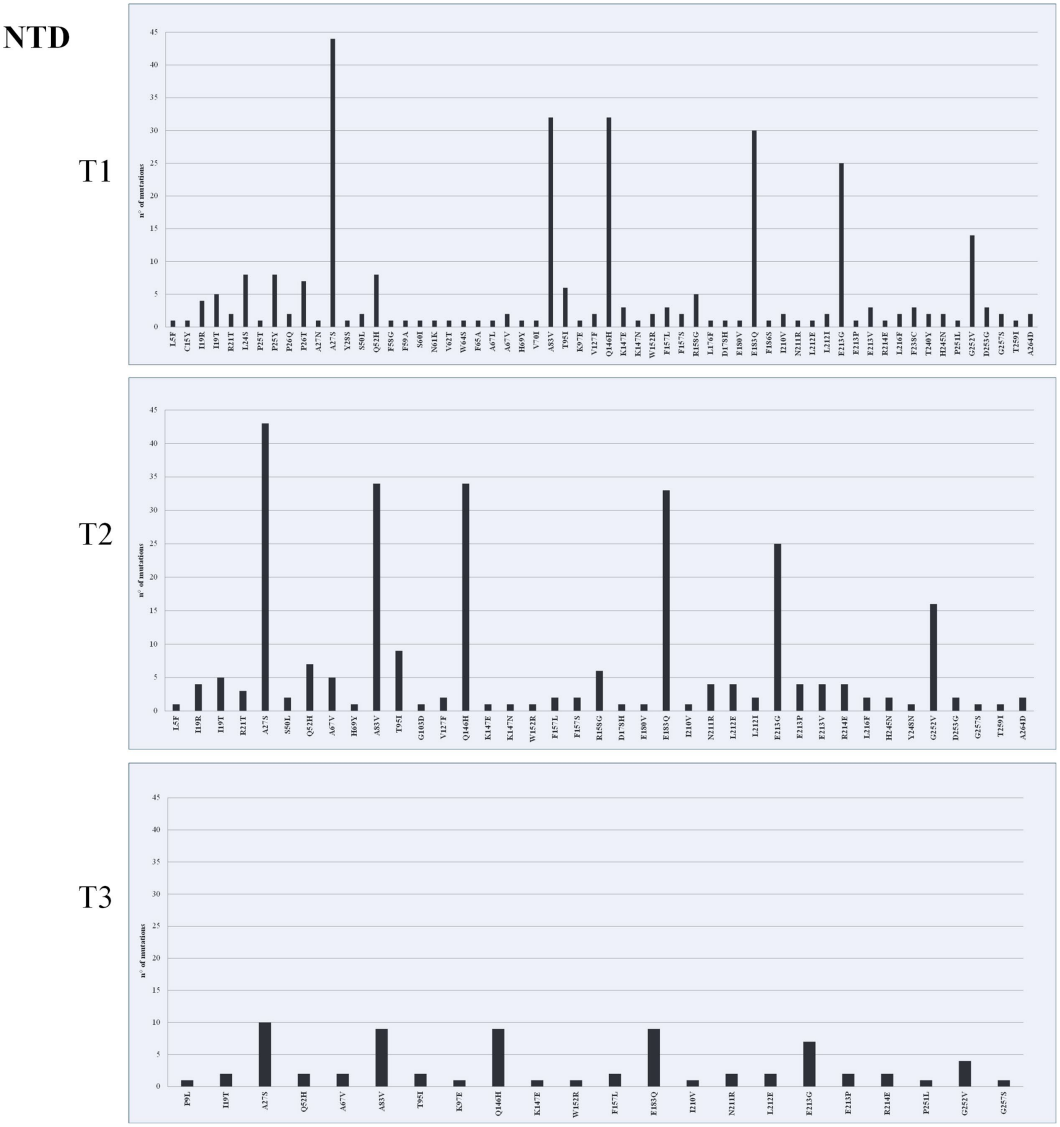
Mutation density of T1, T2, and T3 across N-terminal domain (NTD). The *x* axis shows mutation position and the *y* axis count of mutations.

**Figure 3:**
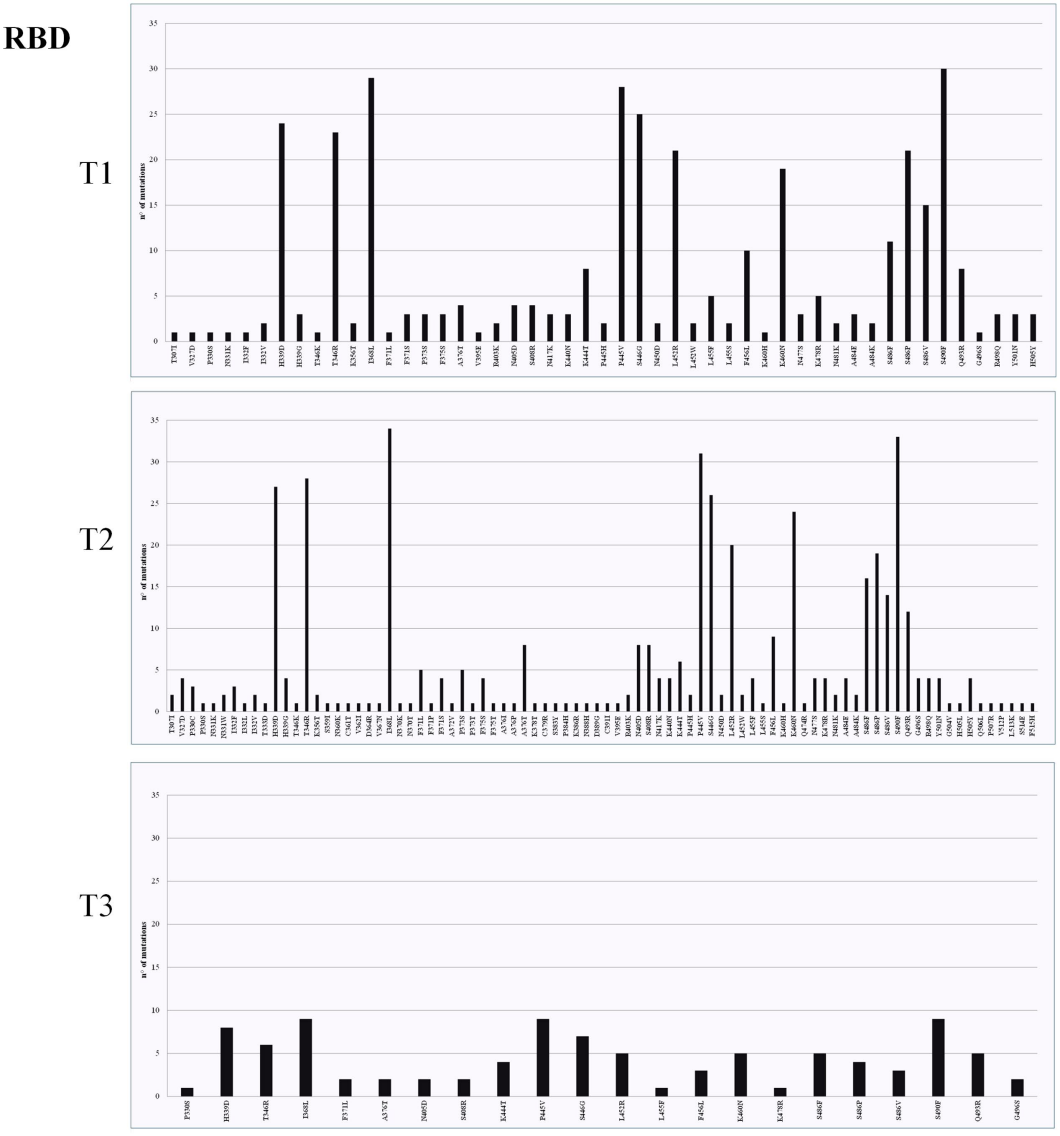
Mutation density of T1, T2, and T3 across receptor-binding domain (RBD). The *x* axis shows mutation position and the *y* axis count of mutations.

**Figure 4:**
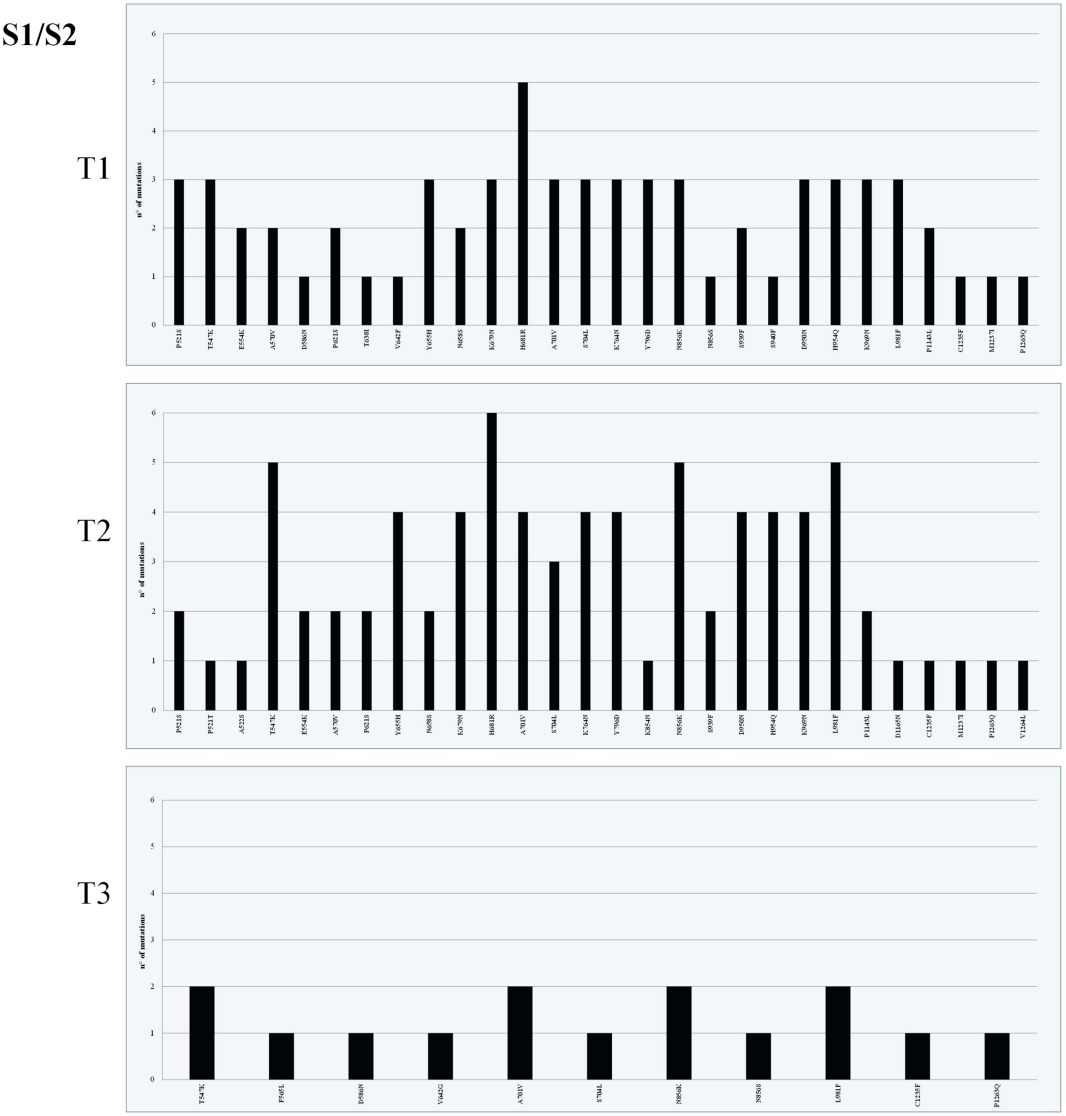
Mutation density of T1, T2, and T3 across subunit 1 (S1) and subunit (S2) domains of the S gene. The *x* axis shows mutation position and the *y* axis count of mutations.

The highest mean of nonsynonymous variant density was observed in the S gene in both T1 (*n* = 1,815) and T2 (*n* = 1,786), specifically in RBD (Figures 2–4). The most frequently found mutations were A27S (*n* = 114), I368L (*n* = 90) and H681R (*n* = 11) in NTD (Figure 2), RBD (Figure 3) and S/S2 regions (Figure 4), respectively. The variant density in the RdRp was 231, corresponding to 38 nonsynonymous mutations within the ORF1b gene in all time points, with a high frequency of P323L (*n* = 113; 48.9%), G671S (*n* = 51; 22.1%), and Y273H (n = 12; 5.2%) mutations. A total of 276 mutations were identified within the PLPro (Papain-like protease) in ORF1a gene. Among these, the G489S (*n* = 92; 33.3%) and T24I (*n* = 91; 32.9%) substitutions were the most represented. The number of mutations identified in the different genes is reported in Supplementary Figure 2.

No drug resistance mutations were observed over time.

Comparing T1 *vs*. T2, 38.8% of subjects increased and 36.7% decreased the number of mutations and 24.5% maintained the same number. Subjects with constant numbers of mutations maintained the same pattern while 55 and 57% of the acquisitions and losses were confirmed and 34% had a combination of acquisition and loss of mutations. Acquired mutations at T2 were not present as minority mutations at T1, 50% of the lost mutations did not persist as minority mutation at T2 (Supplementary Figure 3A).

In T2 *vs*. T3 comparison, 66.7 and 33.3% of subjects increased or maintained the same number of mutations, 75% of subjects with constant number of mutations maintained the same pattern and 63% of the acquisitions were confirmed. 33.3% of the subjects had a combination of acquisition and loss of mutations. New mutations at T3 were not present as minority mutations at T2 (Supplementary Figure 3B).

Comparison of the mutational pattern in the different genes also showed a significant higher overall number of acquired mutations than lost one, in both T1 *vs.* T2 and T2 *vs.* T3 comparisons.

While in T1 *vs.* T2 the acquisitions were prevalent in the S gene and the losses in ORF1a, the opposite was observed in T2 *vs.* T3. Stratifying according to variant, a significant loss of mutations in all genes was observed in the BA.1 variant while in XBB recombinants, acquisition of mutations was significantly higher in ORF1a, ORF1b and S genes compared to N and ORF9b genes. Comparison of the mutational pattern in the different gene portions showed a significant higher overall number of acquired mutations in RDB and NTD of S gene (Table 2).

**Table 2:** Number of acquired/lost mutations in SARS-CoV-2 different genes considering T1 *vs.* T2 (part A) and T2 *vs.* T3 (part B) comparisons.

A	T1 *vs.* T2		B	T2 *vs.* T3	
Gene	Acquisition	Loss	Gene	Acquisition	Loss
ORF1a	53	129	ORF1a	93	0
ORF1b	38	35	ORF1b	13	13
S	200	67	S	11	18
M	2	0	M	1	0
N	7	7	N	1	0
ORF3a	1	0	ORF3a	0	1
ORF6	1	0	ORF6	0	1
ORF8	6	9	ORF8	1	2
ORF7a	0	8	ORF7a	0	0
ORF7b	0	1	ORF7b	0	0
ORF9a	0	0	ORF9a	0	0
ORF9b	0	13	ORF9b	0	0
Total	308	269	Total	120	35

By analyzing different comorbidities, only subjects with cardiovascular disease showed a significant acquisition of mutations compared to losses number of mutations over time (*p = 0.0394*). The majority of acquired mutations were observed in ORF1a (Supplementary Table S3).

No differences were observed when considering different therapies or monotherapy *vs.* combination therapy for SARS-CoV-2.

**Figure 5:**
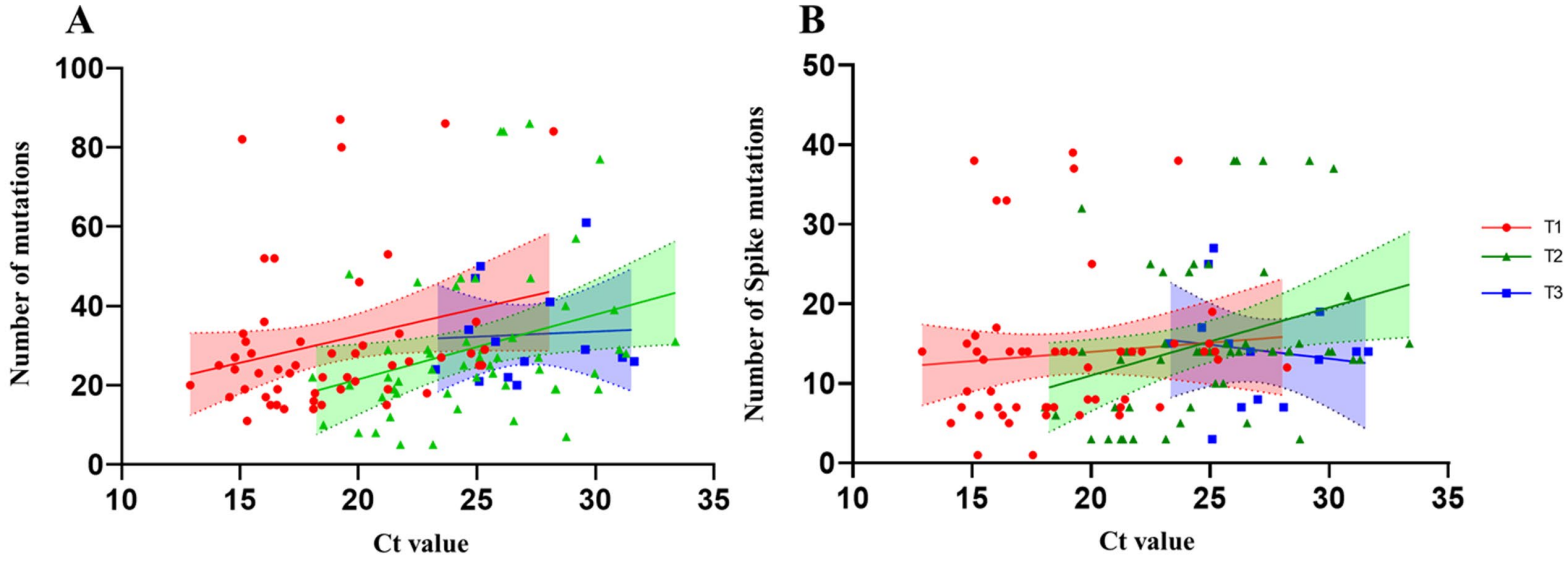
Correlation plots between Ct values and the number of viral mutations. **(A)** Spearman’s correlation tests between Ct values and the total number of mutations along whole genome. T1: (*R*: 0.23, *ρ*: 0.0743); T2: (*R*: 0.36, ρ: 0.0270); T3: (*R*: 0.05, ρ: 0.8556). **(B)** Spearman’s correlation tests between Ct values and the total number of spike mutations. T1: (*R*:0.14, ρ:0.5322); T2: (*R*: 0.36, ρ:0.0230); T3: (*R*: −0.28, ρ:0.6457). Time points are indicated with different symbols and colors as reported in the legend. The *x*-axis displays the Ct values determined by real-time PCR, whereas the *y*-axis represents the number of mutations.

The correlation between the total number of viral mutations and Ct values at three different timepoints (T1, T2, and T3) was evaluated using Spearman’s correlation tests (Figure 5A). At T1, despite a positive trend (r = 0.2293), there was no statistical significance (*p = 0.061*). At T2, a moderate and positive statistically significant correlation was found (r = 0.355; *p = 0.027*), indicating that higher Ct values were associated with a greater number of accumulated viral mutations. At T3, no correlation was observed (r = 0.0528; *p = 0.855*), likely due to the limited sample size at this time point. A similar trend was observed when examining only the Spike protein (Figure 5B) (T1: r = 0.1415, *p = 0.532;* T2, r = 0.3688, *p = 0.023*, and T3: r = −0.2810, *p = 0.645*).

### 3.4 Minority variants

In both T1 *vs.* T2 and T2 *vs.* T3 comparisons, all the confirmed acquisition cases showed *ex novo* mutations. In 4 of 10 cases, lost mutations did not persist as minority mutations at T2. 17 cases presented both acquisition and loss of mutations, of which 12 as *ex novo* mutations or not found in minorities.

In the T2 *vs.* T3 comparison 5 cases presented both acquisition and loss of mutations, of which 3 as *ex novo* mutations or not found in minorities (Supplementary Figure 3).

### 3.5 Phylogenetic analysis of consensus genomes

**Figure 6:**
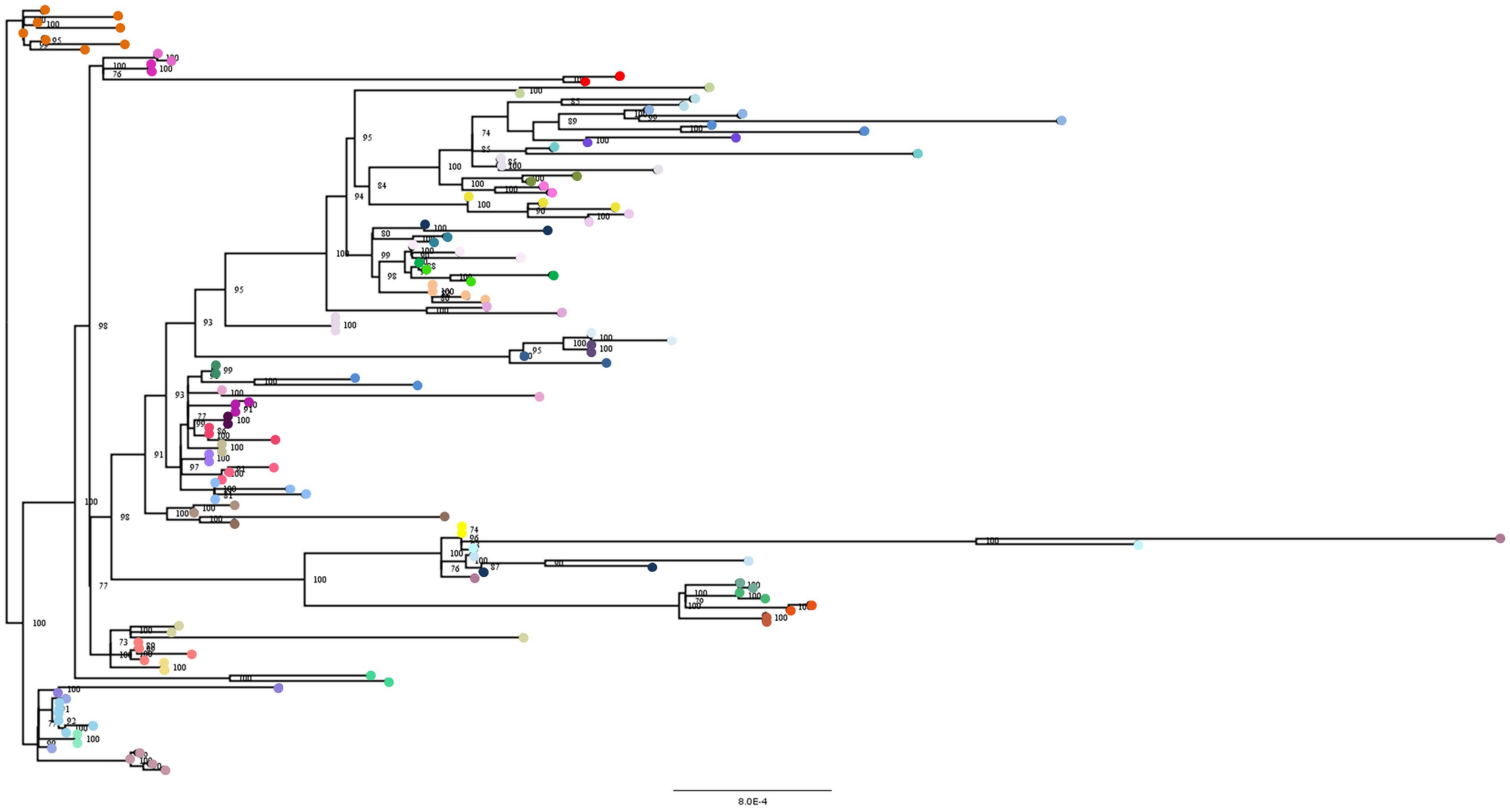
Maximum-likelihood tree of SARS-CoV-2 sequences obtained from studied population. Bootstrap support (>70) is indicated at nodes. Circles at the end of branches are colored different for each patient. Scale bar represents the number of substitutions per site.

The maximum likelihood tree of 139 genomes characterized in this study (58 subjects considered at different time points) is shown in Figure 6. The monophyletic status of the sample derived from the same subjects at different time points was recovered for 49 out of the 58 patients analyzed in this study. No subjects presented a change in variant assignation in different time points included. The majority of 9 strains without monophyletic status were BA.1 (*n* = 4) followed by XBB (*n* = 3), BQ.1 and BA.2 (*n* = 1 each), mainly presented acquired mutations and mixed pattern of mutations.

Based on different collection time of samples not showing monophyletic status we could exclude a reinfection with the same circulating viral variant. Moreover the subjects were hospitalized and in hospital isolation.

## 4 Discussion

The emergence of divergent SARS-CoV-2 lineages has called into question the role of intra host selection in novel variants. Prolonged infections permit additional cycles of viral replication, lead to greater accumulation of intra-host mutations (Kemp et al., 2021; Truong et al., 2021) and allow more time for within-host selection. Few studies that characterize how variants arise and evolve during persistent infections in non-immunocompromised subjects are present (Goldswain et al., 2024; Chaguza et al., 2023; Ghafari et al., 2024), however this information is important for defining the degree with which acute and prolonged infections contribute to global SARS-CoV-2 evolution. In this study, we focused on a high-throughput sequencing from longitudinal samples of 58 patients who remained RT-PCR positive for SARS-CoV-2 for over 3 weeks by performing a viral intra-host genomic analysis. Although the exact biological mechanisms of intra-host population dynamics have yet to be explored; by comparing different time points from the same individual, we were able to track the acquisition and loss of variability during prolonged infection.

Accordingly with other studies, we observed more SNVs at lower viral load; this could be a result of a stochastic sampling as more variants may cross the minimum frequency threshold or can be effect of lower reliability of sequencing (Lythgoe et al., 2021; Voloch et al., 2021; Braun et al., 2021). However, compared to other works on within evolutionary dynamics during acute infection of immunocompetent subjects we observed a higher number of SNVs in all studied time points. This could be due to certain characteristics of studied subjects which had a high median age and a large proportion of subjects with comorbidities requiring hospitalization. Moreover, most of the subjects carried Omicron variants and its recombinant forms, confirming data reporting higher mutation rate in Omicron compared to other variants (Gupta et al., 2024).

Our data suggests the presence of multiple steps in the fitness selection of SARS-CoV-2 mutations.

In the first step occurring in the first week of infection randomized mutations are generated indicating a genetic diversity increase; in the second week a mainly purifying selection process was observed, determining a reduction in the number of mutations. A further increase of novel mutations was observed in subjects with prolonged infection at week three. This mechanism could be further explored in largest population, particularly for T4 and T5 where our analysis was partially conducted.

The fixation of a few mutations and the appearance of previously undetected new ones suggest a combination of selective pressures and a diverse viral population regardless of viral variant and a limited role of minority mutations in predicting viral evolution.

Indeed, only a small subset of minority mutations became dominant in later specimens, not predicting the emergence of mutations in the subsequent periods analysed by setting a cut-off of 5%.

These results suggest that the accumulation of intra-host mutations may be more pronounced during the intermediate phase of infection, coinciding with a progressive decrease in viral load as demonstrated by the association found between number of mutations and Ct values only at T2.

Studies focused on intra-host variability (Pérez-Lago et al., 2022; Dudouet et al., 2022; Avanzato et al., 2020) reported high number of mutations in structural and non-structural proteins such as Spike, RdRp, helicase and PLpro. These findings converge with our results since the most variable regions in our cohort were Spike and ORF1ab.

Among mutations in ORF1b, those in RdRp can contribute significantly to SARS-CoV-2 genome diversity.

A mutation in RdRp that decreases replication fidelity is expected to enhance viral genetic variability, facilitating adaptation to different selective pressures. As reduced fidelity often correlates with elevated replication rates, such mutations could contribute to higher viral titers within infected cells. Particularly, the P323L mutation seems to affect the mutability and possibly transmissibility of SARS-CoV-2 (Eskier et al., 2020). However, no specific conclusion can be drawn in regard to this mutation as it was observed in all subjects.

Helicase, which is encoded by NSP3 region, may play an important role in the prolonged infection. Helicase is a conserved protein responsible for the resolution of RNA secondary structures during the replication cycle of the virus (Jia et al., 2019). Targeting helicase activity using inhibitors could be a potential candidate for COVID-19 therapy (Spratt et al., 2021; Otsuka et al., 2024).

NSP3 portion, which codes PLpro, has been shown to have important functions for host interactions, by ubiquitin-like action on inflammatory response and evasion from type 1 *β*-Interferon response and on viral spreading control (Shin et al., 2020).

Regarding Spike protein, the NTD has a critical role in overall structural conformation of the protein, and mutations occurring in this portion are linked to viral immune escape. Among mutations the A27S was already observed with high frequency (>65%) in different variants (Balech et al., 2025). While the I368L mutation is a recognized substitution in newer variants such as BA.2.86 an JN.1, studies indicate that did not directly cause significant loss of neutralization (Selvavinayagam et al., 2024). The introduction of the H681R mutation in S1-S2 portion is known to restore cell fusion and increases syncytium formation, even in variants that originally showed less of it, such as Omicron (Escalera et al., 2024).

By considering comorbidities, the only significant correlation was found with cardiovascular disease. It is demonstrated that COVID 19 contributes to cardiovascular complications, including acute myocardial injury because of acute coronary syndrome, myocarditis, stress cardiomyopathy, arrhythmias, cardiogenic shock, and cardiac arrest (Kang et al., 2020). It is also demonstrated that ACE-2 is present in cardiomyocytes, implying that the virus directly exerts cardiac cytopathic effects, even in patients with healthy hearts (Cheng et al., 2021). The “cytokine storm,” elicited by a strong activation of the innate immune system, leads to a diffuse endotheliitis and subsequent procoagulant activity and in general to extensive inflammation of the vascular system.

Despite previous works reported that drug resistance mutations observed *in vitro* were rarely found in clinical isolates some works based on longitudinal samples addressed that some treatment such as remdesivir could induce a selective pressure for the virus evolution and viral intra-host diversification (Heyer et al., 2022; Ling-Hu et al., 2025). Nevertheless in the present work no drug-resistant mutations or an increasing frequency of mutations were observed overtime, not even in subjects who received more than one treatment. These results suggest that the selective pressure of drug administration is not necessarily involved in the acquisition of drug-resistant mutations, possibly due to the short duration of treatment and the presence of an acute infection.

Investigating the impact of lineages on the evolutionary patterns of SARS-CoV-2 is highly relevant. Our study encompassed samples collected over an extended period, allowing the inclusion of multiple viral variants ranging from Delta to XBB recombinants. It is well known that XBB presents a strong capacity for crossing over the host immune system, surpassing the immune evasiveness of previous variants (Giancotti et al., 2024). Although this variant is the most prevalent in our study, no association was observed, probably due to the limited size of the population, which did not allow comparison with other variants. The only significant correlation was observed between viral variant and negativization period. This is probably influenced by the interval between the last vaccine administration and infection, although this association was not statistically significant.

Patterns of within-host evolution may differ in individuals with vaccine or infection-induced immunity. In our study no subject has a documented previous infection, but the majority reported a complete cycle of SARS-CoV-2 vaccination and half of the subjects received a first booster dose. Understanding the degree to which intra-host evolution is shaped by vaccine and infection-induced immunity, will be critical for evaluating the mechanism of SARS-CoV-2 evolution.

Finally, by analyzing longitudinal samples, our study enabled the characterization of viral populations over time in individual infections providing enhanced resolution of SARS-CoV-2 mutations dynamics and their frequencies throughout the course of infection.

The present study has several limitations including its single centre nature and limited sample size making it difficult to draw general conclusions regarding mutations frequency in persistent SARS-CoV-2 infection and to compare immunocompromised and non-immunocompromised population. Moreover, the high Ct values made it impossible to obtain complete genome in some cases.

Identification of additional cases of persistent infection are needed to detail the study of the infection dynamics in this diverse population. Understanding the mechanism of virus persistence and eventual clearance will be essential for providing appropriate treatment and preventing transmission of SARS-CoV-2 because persistent infection might occur more frequently not only in immunocompromised patients.

In conclusion, our results highlight that higher intra-host variability is linked with host-pathogen interactions and suggest that about 50% of all patients with COVID-19 develop highly divergent intra patient variants within 3 weeks of infection, regardless of treatment confirming data of other studies indicating that also during acute infections it could be observed the emergence of new viral variants (Farjo et al., 2024; Landis et al., 2023). Enhanced and repeated sequencing-based surveillance of hospitalized COVID-19 patients facilitates the identification of variants of interest and may confer direct clinical as well as broader public health benefits.

## Data Availability

The datasets presented in this study can be found in online repositories. The names of the repository/repositories and accession number(s) can be found in the article/supplementary material.

## Footnotes

1
https://www.protocols.io/view/ncov-2019-sequencing-protocol-v3-locost-bh42j8ye

2
https://www.gisaid.org/

3
https://pangolin.cog-uk.io/

4
https://clades.nextstrain.org/

5
https://covdb.stanford.edu

6
https://www.megasoftware.net/

7
http://www.iqtree.org/
