# Supplementary material for: SARS-CoV-2 intra-host evolution during acute infection in COVID-19 patients
Source: Front Microbiol. 2026 Apr 28;17:1794039. doi: 10.3389/fmicb.2026.1794039 (PMC13161087; doi:10.3389/fmicb.2026.1794039)
Supplement: Supplementary file 1 [file Data_Sheet_1.pdf]

# *Supplementary Material*

## 1 Supplementary Figures and Tables

### 1.1 Supplementary Figures

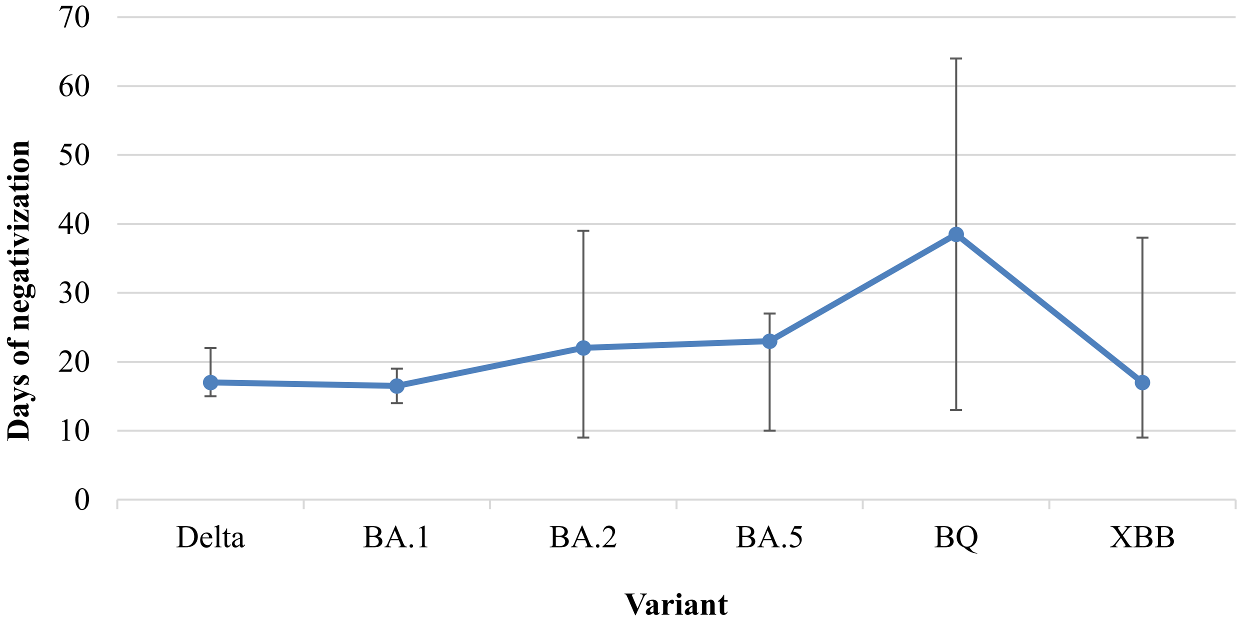


**Supplementary Figure1**. Days of negativization according to viral variant.

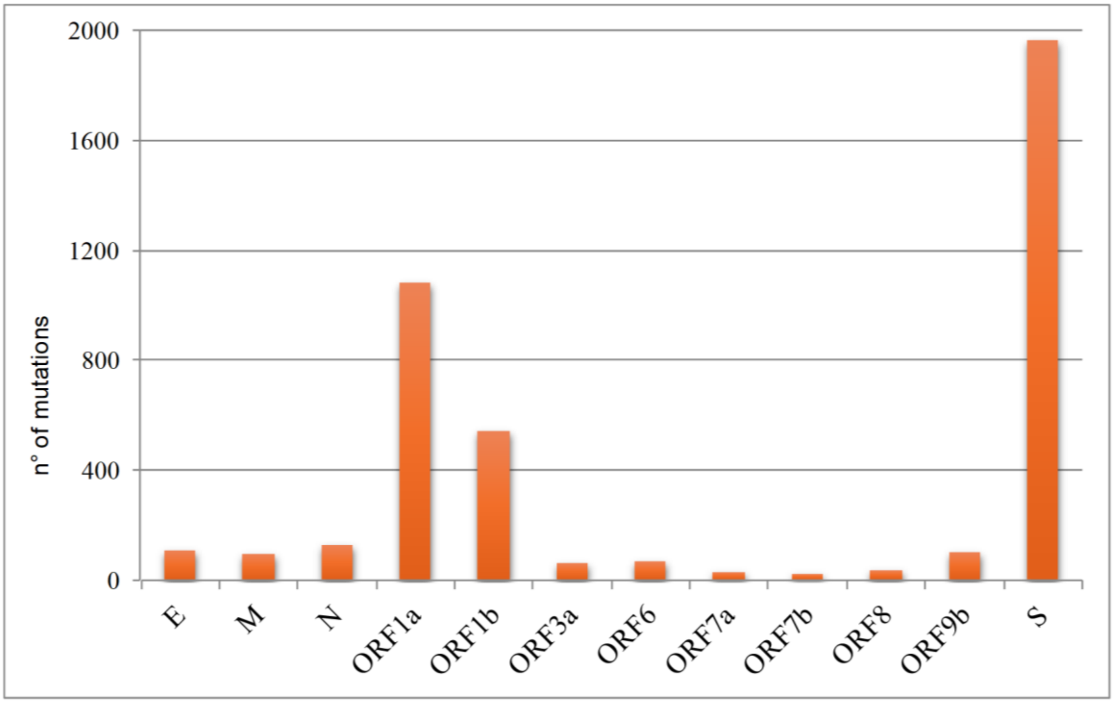


**Supplementary Figure2.** Count of mutations per gene. The x axis shows gene portion and the y axis count of mutations.

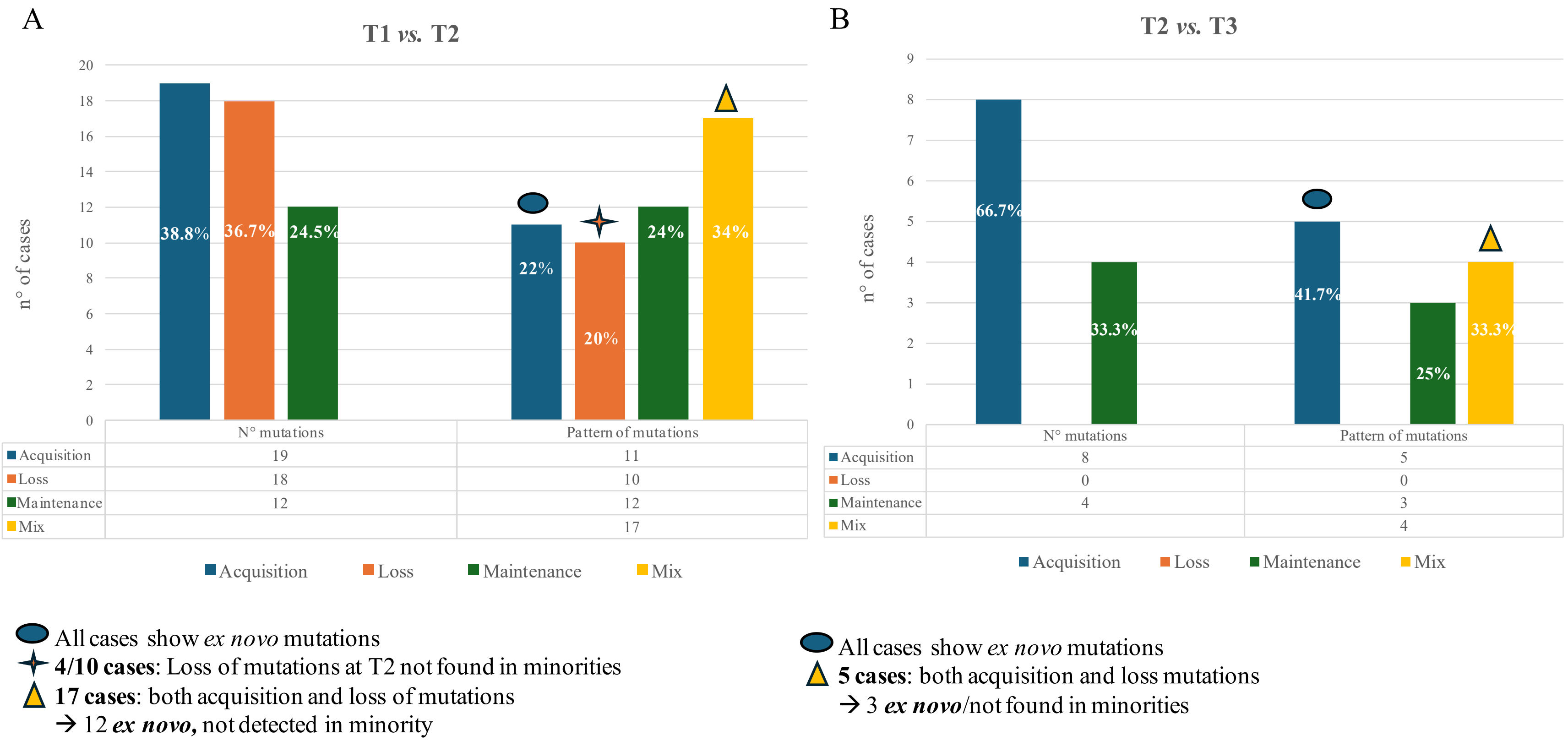


| | N° mutations | Pattern of mutations |
|---|---|---|
| Acquisition | 19 | 11 |
| Loss | 18 | 10 |
| Maintenance | 12 | 12 |
| Mix | | 17 |

| | N° mutations | Pattern of mutations |
|---|---|---|
| Acquisition | 8 | 5 |
| Loss | 0 | 0 |
| Maintenance | 4 | 3 |
| Mix | | 4 |

**Supplementary Figure3**. Comparison between T1 *vs.* T2 and T2 *vs.* T3 in terms of number of mutations and mutational pattern.

## 1.2 Supplementary Tables

**Supplementary Table S1.** Comorbidities found in the study population.

| Comorbidities | Total (n = 58) |
|---|---|
| Pneumonia | 41 (71.9) |
| *missing* | 1 |
| Respiratory failure | 36 (63.2) |
| *missing* | 1 |
| Pulmonary disease | 20 (35.1) |
| *missing* | 1 |
| Rheumatoid factor | 8 (14.0) |
| *missing* | 1 |
| Oncological pathology | 15 (26.3) |
| *missing* | 1 |
| Diabetes | 23 (40.4) |
| *missing* | 1 |
| Cardiovascular diseases | 39 (68.4) |
| *missing* | 1 |
| Nephrological pathology | 12 (21.1) |
| *missing* | 1 |
| Onco-hematological pathology | 8 (14.0) |
| *missing* | 1 |
| Immunoiatrogenic | 2 (3.5) |
| *missing* | |
| HIV | 2 (3.5) |
| *missing* | 1 |
| Hepatitis | 2 (3.5) |
| *missing* | 2 |

**Supplementary Table S2.** Accession numbers of samples studied.
* patient with cardiovascular disease.

| ID | EPI_ISL |
|---|---|
| 29_T1 | EPI_ISL_16979865 |
| 2_T1* | EPI_ISL_20262850 |
| 30_T1* | EPI_ISL_18863859 |
| 31_T1 | EPI_ISL_18643047 |
| 32_T1* | EPI_ISL_18863856 |
| 33_T1 | EPI_ISL_20262851 |
| 34_T1* | EPI_ISL_16979864 |
| 35_T1* | EPI_ISL_20263235 |
| 36_T1* | EPI_ISL_17502340 |
| 3_T1* | EPI_ISL_20262852 |
| 37_T1* | EPI_ISL_16979859 |
| 4_T1* | EPI_ISL_20262853 |
| 38_T1* | EPI_ISL_20263236 |
| 39_T1* | EPI_ISL_18863864 |
| 5_T1 | EPI_ISL_20262854 |
| 41_T1* | EPI_ISL_18643033 |
| 6_T1* | EPI_ISL_20262855 |
| 42_T1 | EPI_ISL_17502341 |
| 43_T1 | EPI_ISL_17502342 |
| 7_T1* | EPI_ISL_20262856 |
| 45_T1* | EPI_ISL_16979830 |
| 8_T1 | EPI_ISL_9221296.1 |
| 46_T1 | EPI_ISL_20262857 |
| 47_T1* | EPI_ISL_18643027 |
| 48_T1* | EPI_ISL_18863868 |
| 9_T1 | EPI_ISL_20262858 |
| 50_T1* | EPI_ISL_18863869 |
| 10_T1* | EPI_ISL_20262859 |
| 11_T1* | EPI_ISL_20262860 |
| 51_T1* | EPI_ISL_18643030 |
| 12_T1* | EPI_ISL_20262861 |
| 13_T1 | EPI_ISL_20263237 |
| 52_T1* | EPI_ISL_16907047 |
| 54_T1* | EPI_ISL_16907058 |
| 16_T1* | EPI_ISL_20262862 |
| 55_T1 | EPI_ISL_17502348 |
| 56_T1 | EPI_ISL_18643023 |
| 57_T1* | EPI_ISL_18643038 |
| 58_T1* | EPI_ISL_16907038 |
| 17_T1* | EPI_ISL_20262863 |
| 18_T1 | EPI_ISL_20262864 |
| 59_T1* | EPI_ISL_18643045 |
| 60_T1* | EPI_ISL_20263238 |
| 19_T1 | EPI_ISL_20263239 |
| 61_T1* | EPI_ISL_18643024 |
| 62_T1* | EPI_ISL_18286017 |
| 21_T1 | EPI_ISL_20263240 |
| 63_T1* | EPI_ISL_20262865 |
| 64_T1 | EPI_ISL_18863880 |
| 65_T1 | EPI_ISL_20262866 |
| 66_T1* | EPI_ISL_18863879 |
| 68_T1* | EPI_ISL_16913050 |
| 69_T1 | EPI_ISL_16979846 |
| 70_T1* | EPI_ISL_17502349 |
| 23_T1* | EPI_ISL_20262867 |
| 24_T1 | EPI_ISL_20262868 |
| 25_T1* | EPI_ISL_20263241 |
| 26_T1* | EPI_ISL_20262869 |

**Supplementary Table S3.** Specific mutations in subjects with cardiovascular complications.

| Gene | Timepoint | | | | |
|---|---|---|---|---|---|
| | T1 | T2 | T3 | T4 | T5 |
| E | L21F | L21F | L21F | - | G96V |
| N | G30A, A208V, K347T, Q418L | A208V, K347T, Q418L | A208V, G238S | Q418L | Q418L |
| ORF1a | S142L, T170I, L204I, R207C, R301L, K322R, Q455T, K456E, G519S, E525V, Q526H, L590F, P777S, C800V, A801L, P820T, S944L, Q1198H, Q1198K, E1205D, S1221L, M1448I, P1449F, T1543I, M1586T, T1678N, L1683F, L1725E, Q1800V, E1801L, S1802T, P1803S, V1887I, G2091S, N2111T, E2112K, L2113T, R2159W, C2180G, G2207S, K2219E, S2242F, A2279V, S2500F, S2556L, V3078A | T170I, L204I, R207C, R301L, K322R, K367T, N418S, G519S, L590F, C655R, D691N, P777S, S944L, P959S, G993S, Q1198H, Q1198K, S1221L, T1543I, M1586T, T1678N, L1683F, L1725D, L1748V, V1751E, T1754A, C1755G, T1788K, K1791E, Q1792K, Q1792N, A1793D, A1793L, T1794K, Y1796E, L1797G, L1797K, Q1799K, Q1799V, Q1800L, Q1800V, E1801L, E1801T, S1802T, P1803S, S1857L, V1887I, G2091S, A2129V, N2140I, R2159W, C2180G, T2183H, S2185C, T2186F, G2207S, S2242F, A2279V, S2556L, P3096W, G3103Y | A117T, S142L, R301L, C370F, H388Y, D629N, G993S, Q1198K, E1205D, S1221L, T1721P, G1723R, E1724A, D1727N, E1730D, S1733C, Y1734H, L1735M, L1735T, F1736I, F1736M, Q1737H, H1738L, N1740H, L1741F, D1742Y, T1754I, Q1758P, Q1759L, T1760S, K1763M, G1764R, T1773R, Q1778K, K1780L, I1785M, C1787G, K1791N, Q1792S, T1794R, K1795S, Q1800K, P1803K, F1804C, M1806F, M1806F, S1857L, R2159W, S2242F, I2426T, V2427R, S2500V, I2501K, H2516Y, R2875C, | S142L, N418S, C655R, P959S, G1073R, P1158S, E1205D, N2498H | V111L, S142L, P193L, N418S, W423E, V424K, P425A, R426A, A427I, S428G, A429K, T436A, V438D, T614I, C655R, P959S, G993S, G1073R, P1158S, E1205D, T1754I, R2159W, T2335N, R2336M, I2346L, S2352L, N2361T, V2400F, V2401F, D2402F, G2403C, L3101F, P3102V, G3103S |
| ORF1b | P85S, N480K, N487T, G494N, G977S, G1014T, K1017L, T1555I, V1607I, V1706I, D1746Y, Y1944P, S2198I, Q2425R, R2483K, K2557R, I2566L, K2567L | P85S, Q435P, D436S, G437P, N438R, A439K, E513S, Q515D, A616T, D901G, T1050I, E1051S, R1052A, L1053A, L1055S, T1404M, T1555I, M1582S, G1583L, V1607I, S1658F, K1663L, P1664K, P1665L, P1666F, V1706I, D1746Y, N1946S, S2198I, Q2425R, R2483K, K2557R | K150R, A186T, V426I, D514T, A517S, L518F, D901G, T1404M, V1706I, S2198I, P2204S, Q2425R, K2557R | D901G, T1404M, T1453I, R1802T, L1804Q, P1821K | H716Y, D901G, H998N, T1404M, T1453I, C1535F, S1702F, D1797Y, K1873R, E2221L |
| ORF3a | A59V, L86F, I123T, L140F | A59V, L86F, L140F | - | I123T | I123T |
| ORF6 | N39D | N39D | - | - | - |
| ORF7a | L12F, A13V, F46L, H47P, P48S, L49Y, K117L, R118F, | L12F, A13V | - | - | - |
| ORF7b | L14S, A43L | L14S, A43L | - | - | |
| ORF8 | I58T | S54V, P56L, I58T | Q18G | - | I71T |
| ORF9b | E27Q, N55S, T60I | N55S, T60I | | - | V93L |
| S | A27N, Y28S, A67L, K97E, K147E, K147N, W152R, F157L, E180V, I210V, P251L, G257S, T307I, K460H, K478R, P521S, A701V, N856S, M1237I, P1263Q | G103D, K147E, K147N, W152R, F157L, E180V, I210V, Y248N, G257S, T307I, P330C, P330S, N331W, I332L, T333D, S359I, N360K, C361T, V362I, D364R, V367N, N370K, N370T, F371P, A372V, P373S, F375T, A376I, A376P, K378T, C379R, S383Y, P384H, K386R, N388H, D389G, C391I, K460H, Q474R, K478R, G504V, H505L, [illegible] | P9L, K97E, K147E, W152R, F157L, I210V, P251L, G257S, P330S, D586N, A701V, N856S, P1263Q | K97E, Y248N, P251L, P330S | P9L, K97E, Y248N, P251L |
